# Supplementary figures and images for: Temperature sensitive contact modes allosterically gate TRPV3
Source: PLoS Comput Biol. 2023 Oct 13;19(10):e1011545. doi: 10.1371/journal.pcbi.1011545 (PMC10599574; doi:10.1371/journal.pcbi.1011545)

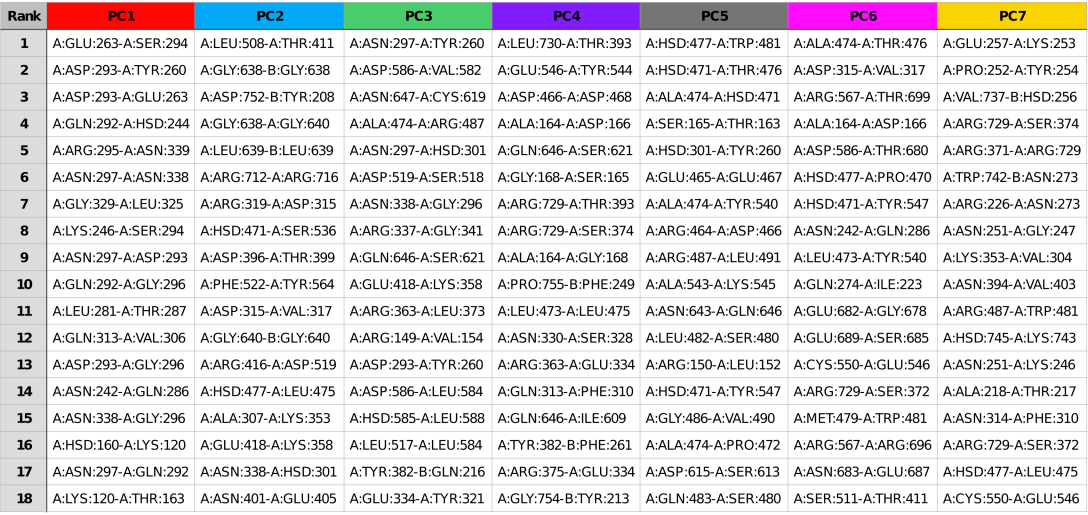

Supplement: S1 Table — (TIFF) [file pcbi.1011545.s001.tiff]

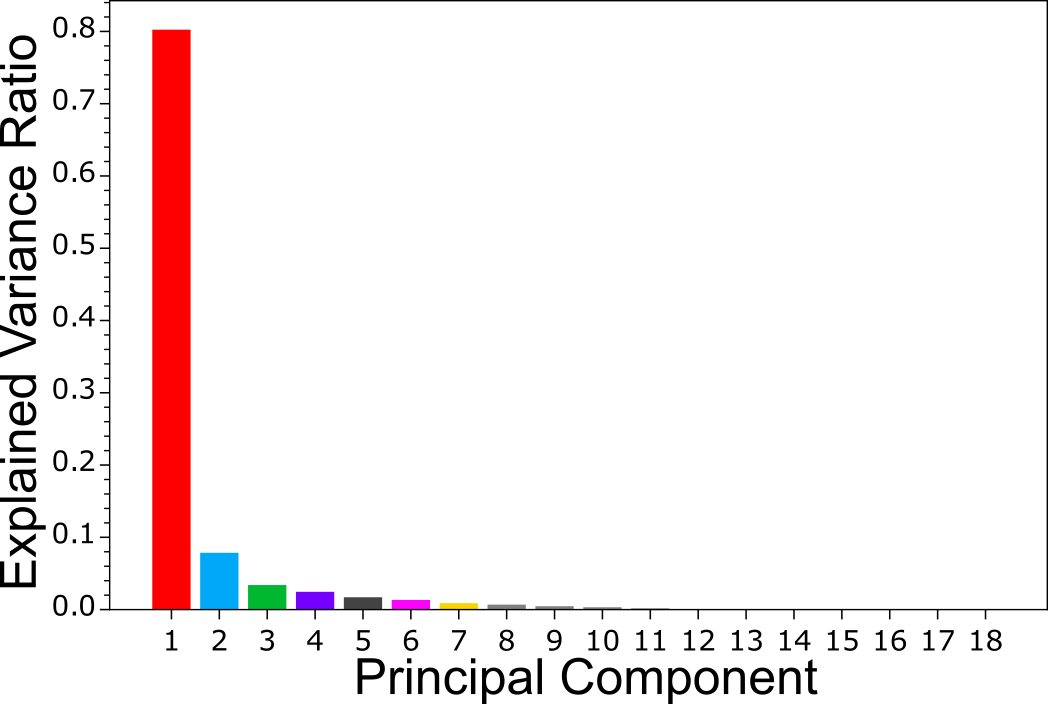

Supplement: S1 Fig — PCs 1–7 were included in the analysis based on the difference of roots significance test. (TIFF) [file pcbi.1011545.s002.tiff]

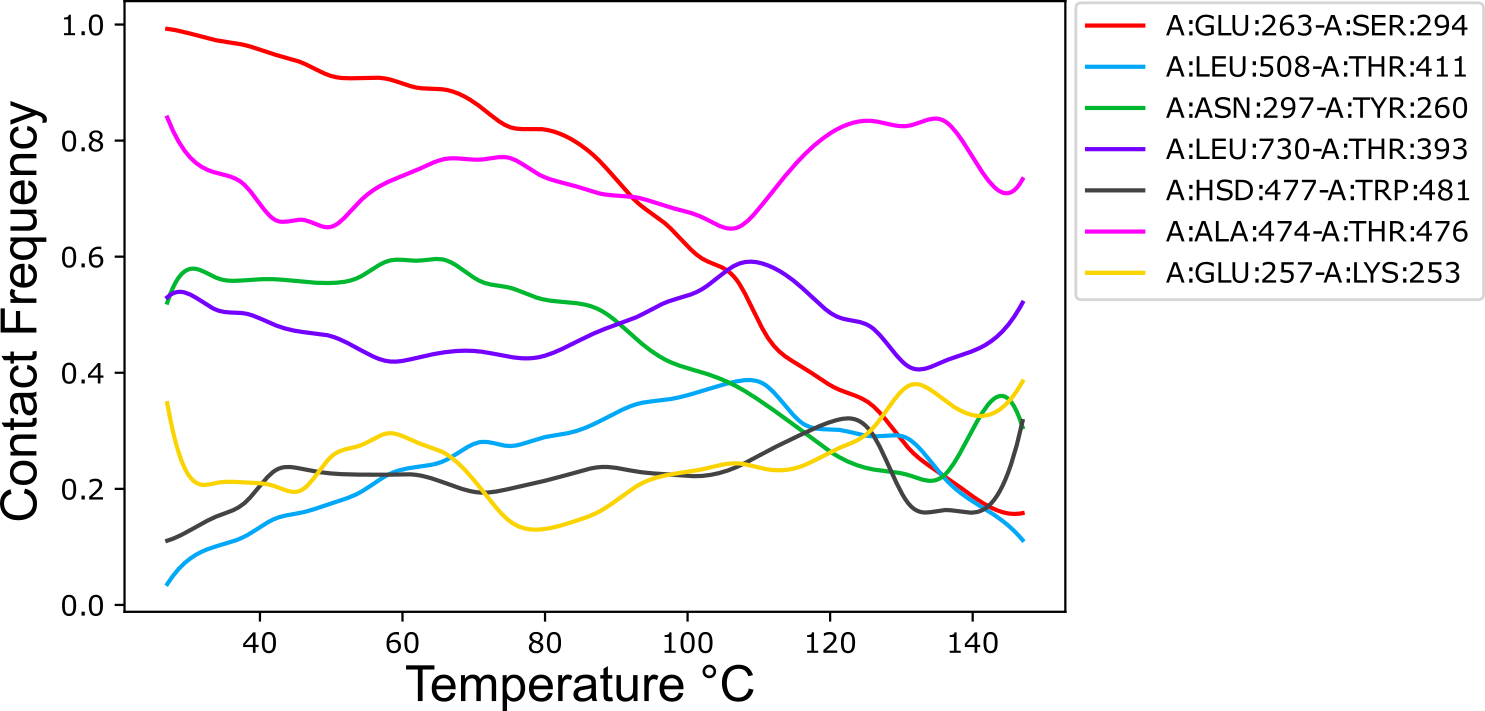

Supplement: S2 Fig — These illustrate that the highest ranked contacts on each PC follow a trend that resembles the projections in Fig 2B. If they follow an opposite trend as the projection, they are inversely correlated on the PC. (TIFF) [file pcbi.1011545.s003.tiff]

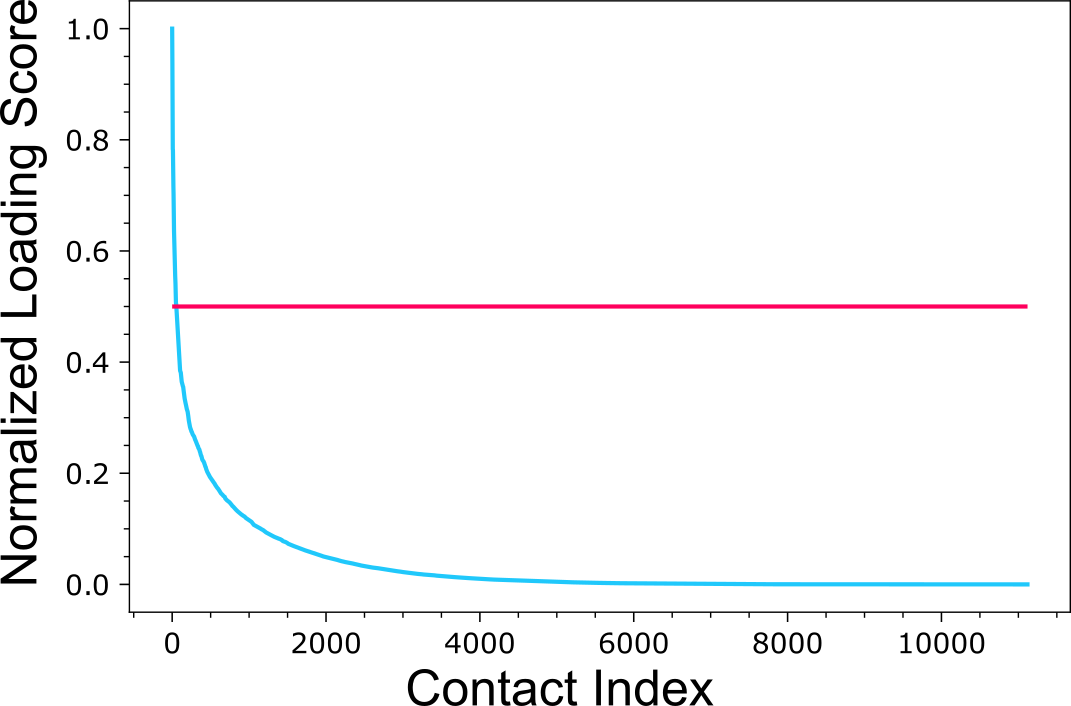

Supplement: S3 Fig — For all 7 of the PCs analyzed, all of the highest ranked contacts (top 18) have loading scores above 0.5 (horizontal cutoff bar). (TIFF) [file pcbi.1011545.s004.tiff]

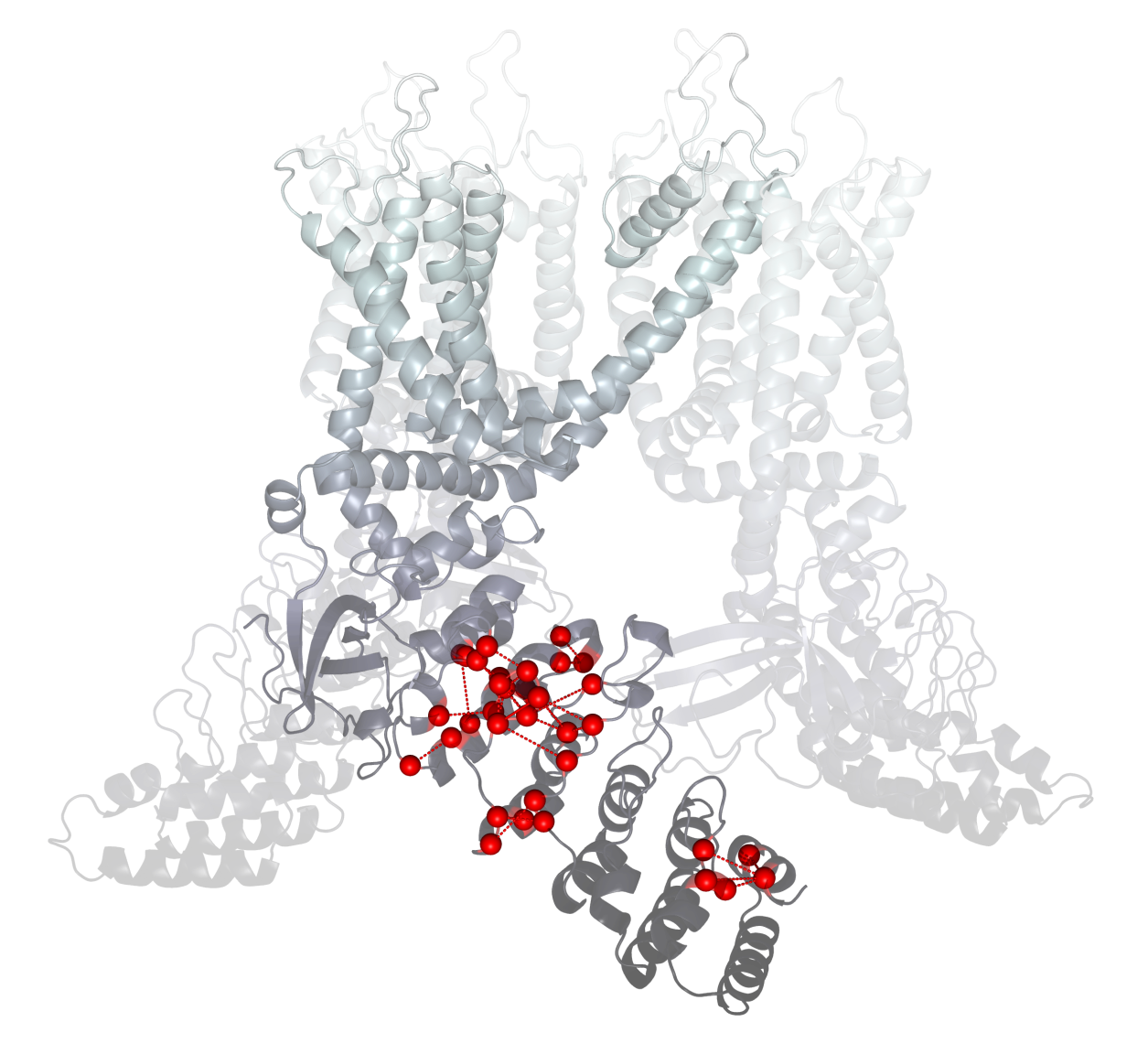

Supplement: S4 Fig — (TIFF) [file pcbi.1011545.s005.tiff]

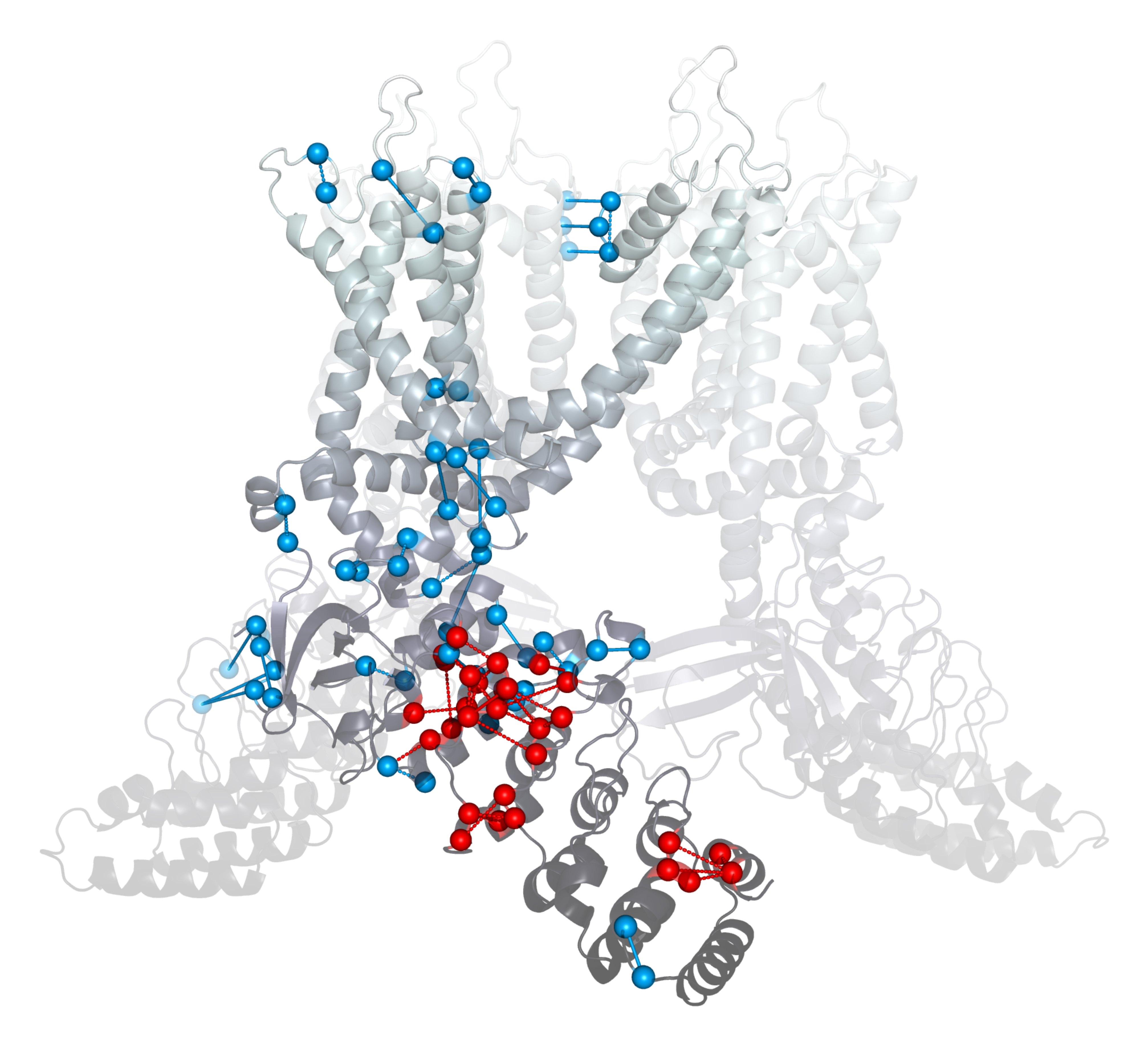

Supplement: S5 Fig — (TIFF) [file pcbi.1011545.s006.tiff]

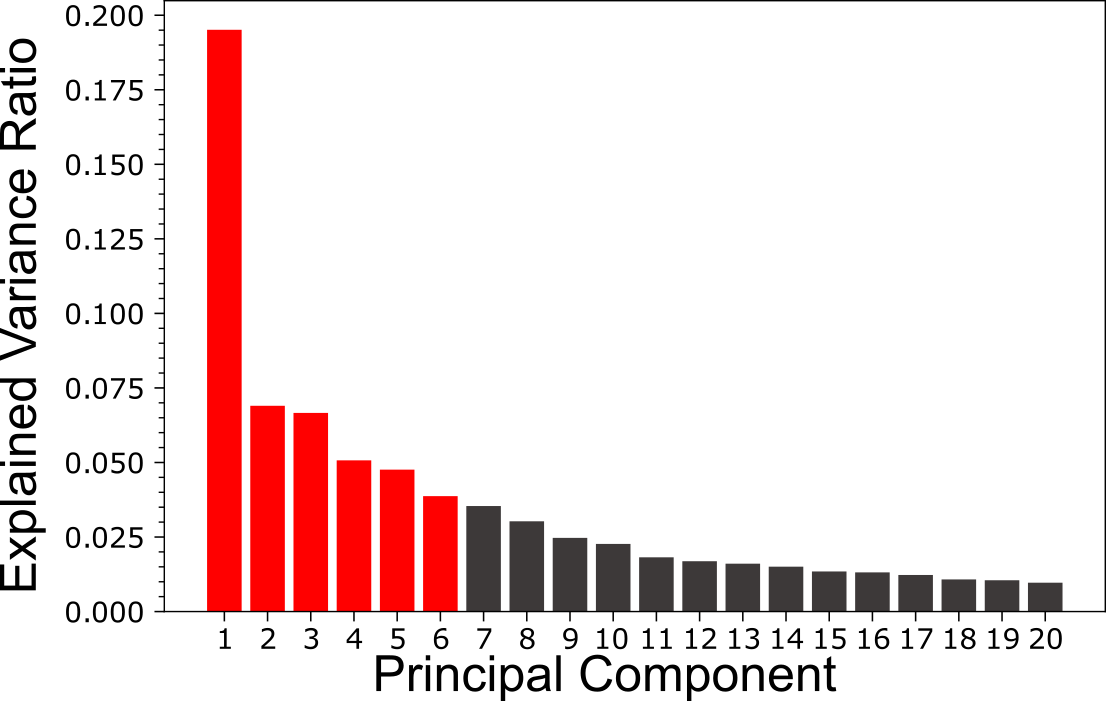

Supplement: S6 Fig — The structures in Figs 3 and S7 are taken from the first six (red) PCs which explain approximately 50% of the variance. (TIFF) [file pcbi.1011545.s007.tiff]

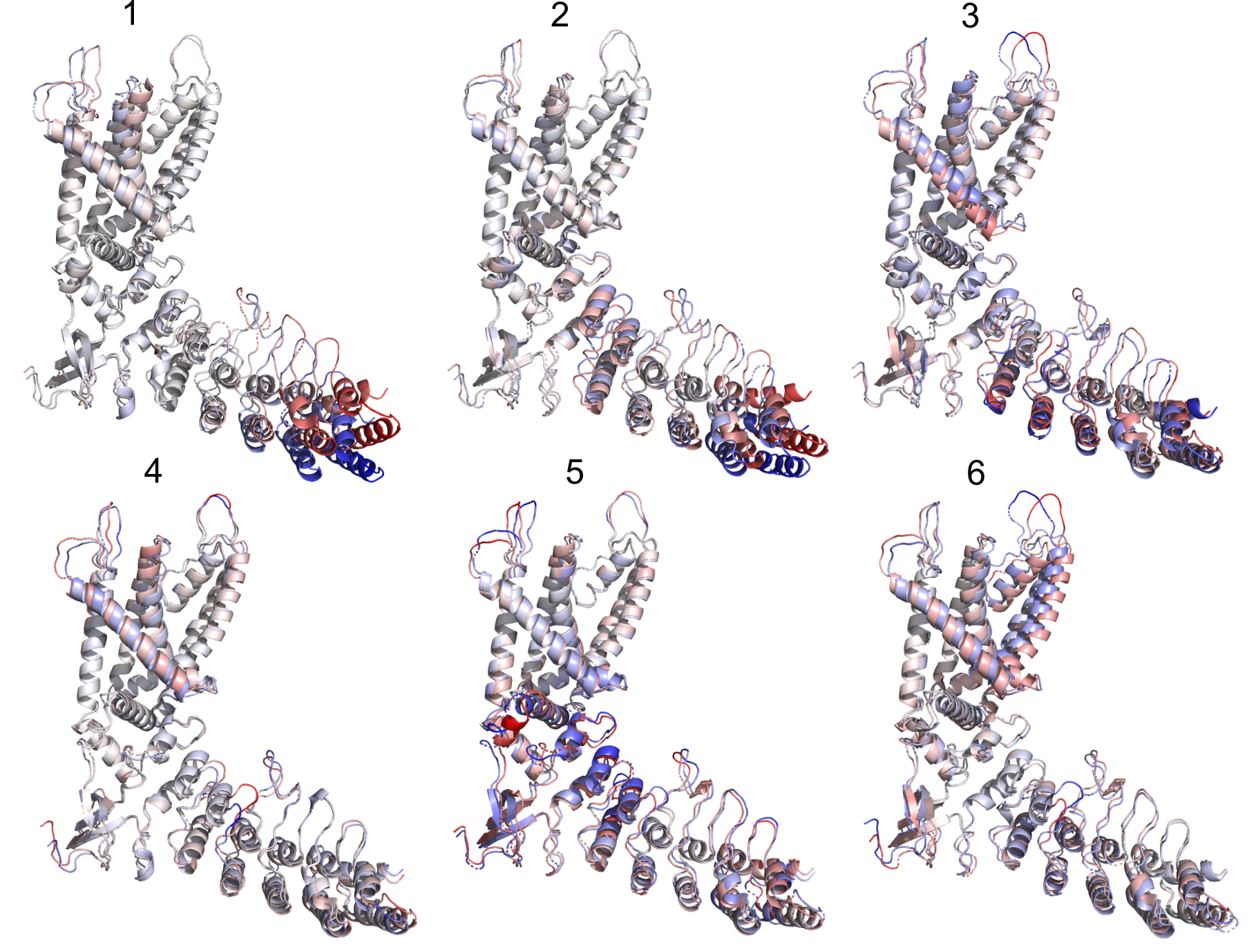

Supplement: S7 Fig — Residues experiencing the most displacement on the PC have the most saturated color. (TIFF) [file pcbi.1011545.s008.tiff]

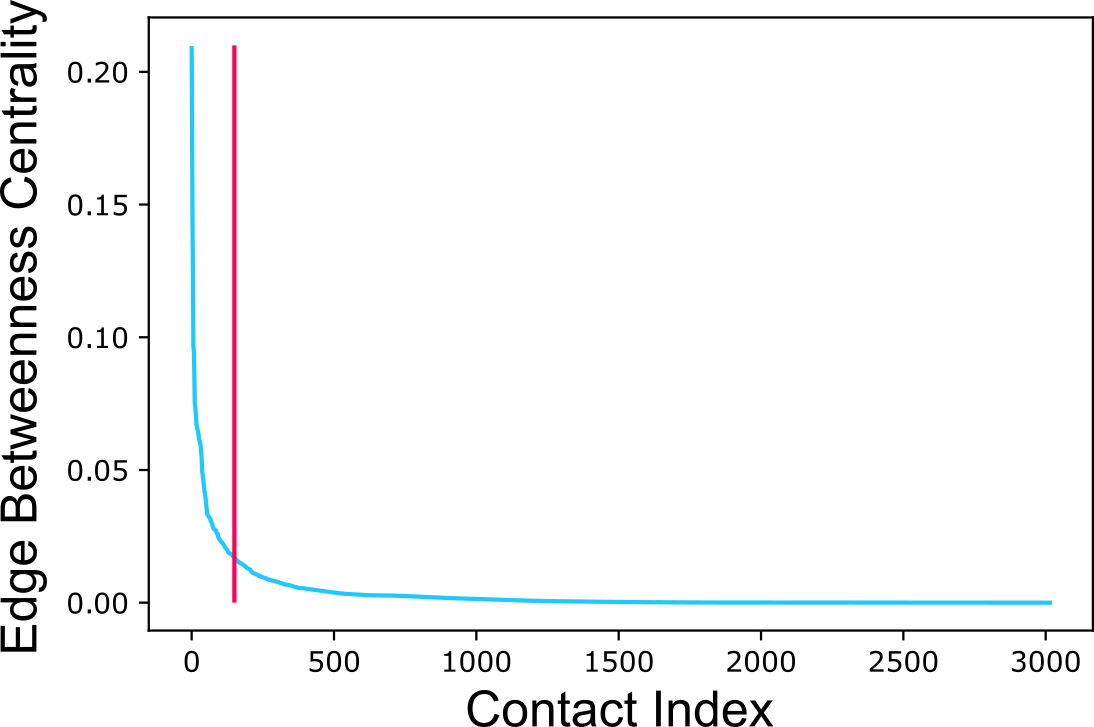

Supplement: S8 Fig — Fig 4C and 4D depict only the most central edges left of the vertical red cutoff bar. (TIFF) [file pcbi.1011545.s009.tiff]

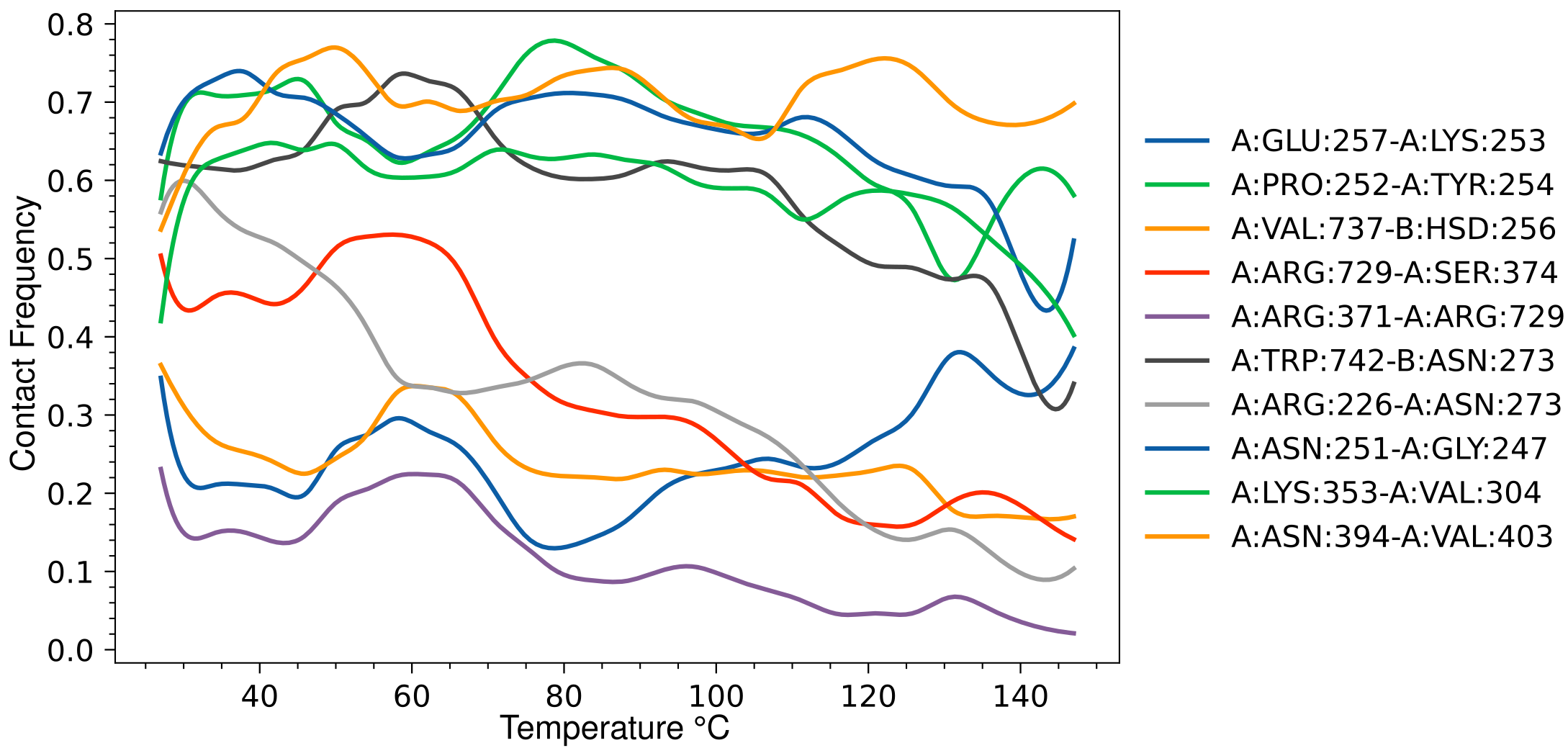

Supplement: S9 Fig — Most of these occur around the IPCD (beta sheet and finger 3 loop of adjacent protomers). The noticeable bump centered around 60°C is of a similar magnitude to the change in expanded gate probability in the same temperature range. (TIFF) [file pcbi.1011545.s010.tiff]

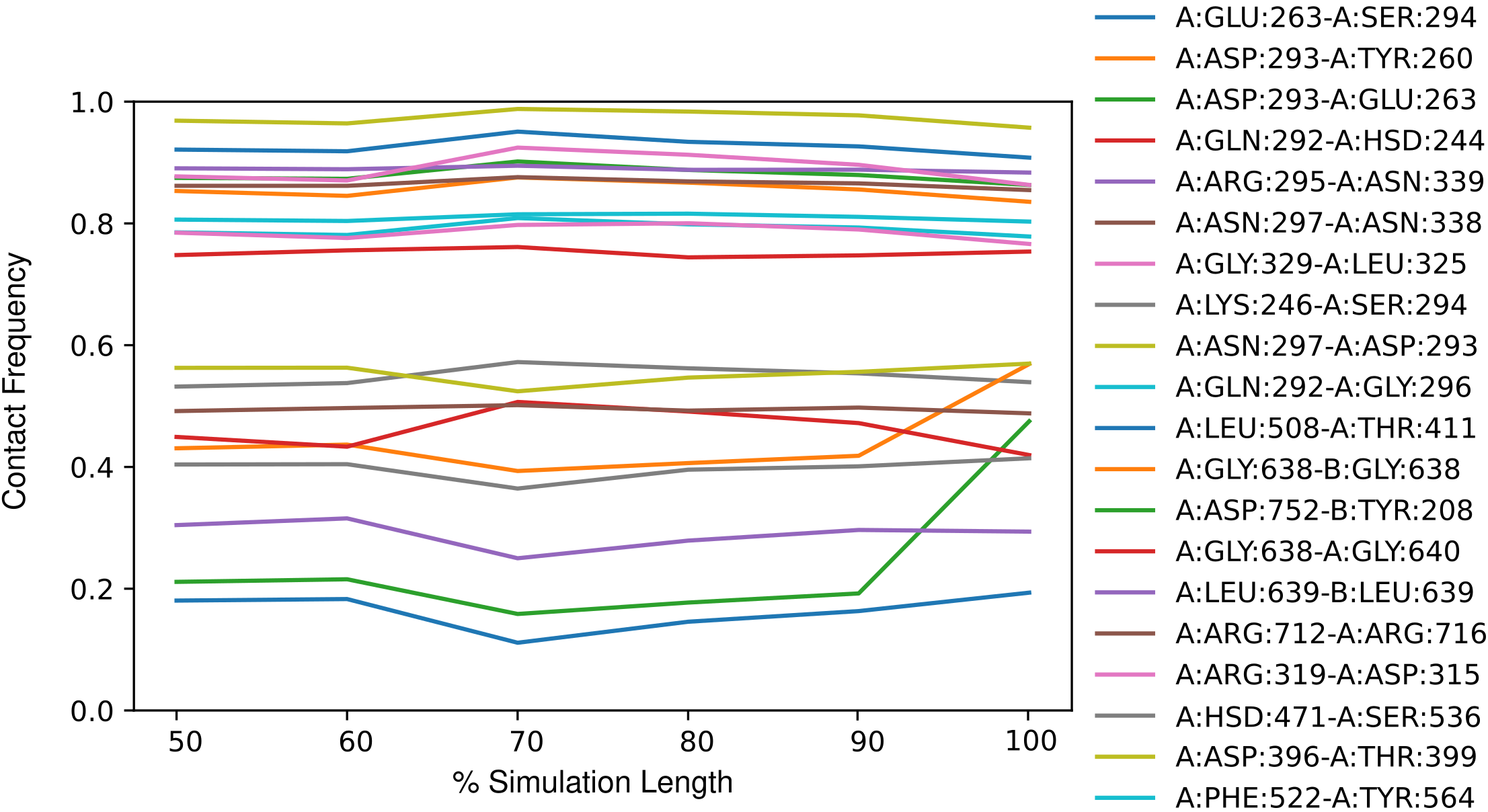

Supplement: S10 Fig — (TIFF) [file pcbi.1011545.s011.tiff]
